# Dichlorido{*N*,*N*-dimethyl-*N*′-[1-(pyridin-2-yl)ethyl­idene]ethane-1,2-diamine-κ^3^ *N*,*N*′,*N*′′}cadmium

**DOI:** 10.1107/S1600536811005538

**Published:** 2011-02-19

**Authors:** Nura Suleiman Gwaram, Hamid Khaledi, Hapipah Mohd Ali

**Affiliations:** a Department of Chemistry, University of Malaya, 50603 Kuala Lumpur, Malaysia

## Abstract

In the title compound, [CdCl_2_(C_11_H_17_N_3_)], the Schiff base acts as an *N*,*N*′,*N*′′-tridentate ligand towards the Cd^II^ ion. Two Cl atoms complete a distorted square-pyramidal geometry around the metal atom. In the crystal, a C—H⋯Cl inter­action connects pairs of mol­ecules into centrosymetric dimers.

## Related literature

For the structure of a CuCl_2_ complex of the same Schiff base, see: Saleh Salga *et al.* (2010 ▶). For the structure of a similar Cd^II^ complex, see: Bian *et al.* (2003 ▶). For a description of the geometry of complexes with five-coordinate metal atoms, see: Addison *et al.* (1984 ▶).
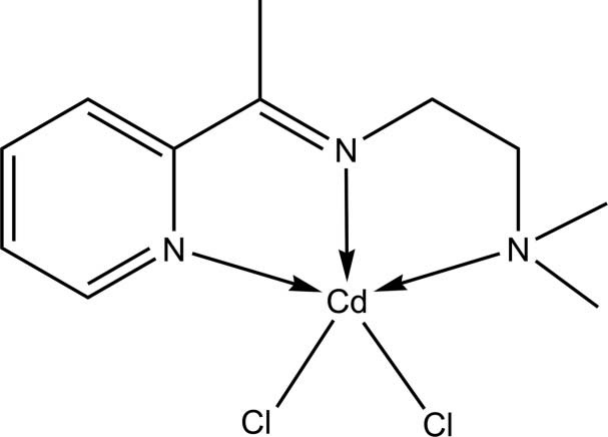


## Experimental

### 

#### Crystal data

[CdCl_2_(C_11_H_17_N_3_)]
*M* *_r_* = 374.58
Triclinic, 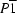

*a* = 8.0276 (2) Å
*b* = 9.6048 (2) Å
*c* = 10.0851 (2) Å
α = 102.534 (1)°
β = 103.365 (1)°
γ = 97.850 (1)°
*V* = 724.09 (3) Å^3^
*Z* = 2
Mo *K*α radiation
μ = 1.86 mm^−1^
*T* = 100 K
0.23 × 0.11 × 0.04 mm

#### Data collection

Bruker APEXII CCD diffractometer
Absorption correction: multi-scan (*SADABS*; Sheldrick, 1996 ▶) *T*_min_ = 0.674, *T*_max_ = 0.929
5023 measured reflections
2652 independent reflections
2547 reflections with *I* > 2σ(*I*)
*R*_int_ = 0.014

#### Refinement

*R*[*F* ^2^ > 2σ(*F* ^2^)] = 0.016
*wR*(*F* ^2^) = 0.038
*S* = 1.08
2652 reflections
157 parameters
H-atom parameters constrained
Δρ_max_ = 0.29 e Å^−3^
Δρ_min_ = −0.39 e Å^−3^

### 

Data collection: *APEX2* (Bruker, 2007 ▶); cell refinement: *SAINT* (Bruker, 2007 ▶); data reduction: *SAINT*; program(s) used to solve structure: *SHELXS97* (Sheldrick, 2008 ▶); program(s) used to refine structure: *SHELXL97* (Sheldrick, 2008 ▶); molecular graphics: *X-SEED* (Barbour, 2001 ▶); software used to prepare material for publication: *SHELXL97* and *publCIF* (Westrip, 2010 ▶).

## Supplementary Material

Crystal structure: contains datablocks I, global. DOI: 10.1107/S1600536811005538/pv2387sup1.cif

Structure factors: contains datablocks I. DOI: 10.1107/S1600536811005538/pv2387Isup2.hkl

Additional supplementary materials:  crystallographic information; 3D view; checkCIF report

## Figures and Tables

**Table 1:** Hydrogen-bond geometry (Å, °)

*D*—H⋯*A*	*D*—H	H⋯*A*	*D*⋯*A*	*D*—H⋯*A*
C7—H7*B*⋯Cl2^i^	0.98	2.77	3.679 (2)	155

Symmetry code: (i) 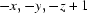.

**Fig. 1.**
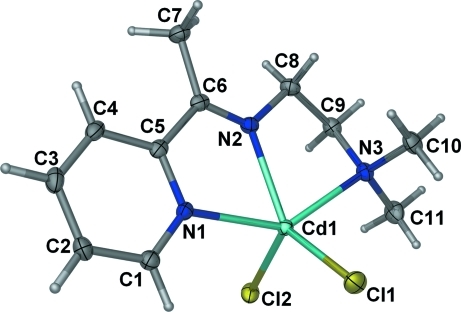
Thermal ellipsoid plot of the title compound at the 50% probability level. Hydrogen atoms are drawn as spheres of arbitrary radius.

## supplementary crystallographic information

### Comment

The title compound was obtained *via* the complexation of CdCl_2_ by *N,N*-dimethyl-*N'*-[methyl(2-pyridyl)methylene]ethane-1,2-diamine. Similar to the structure of the analogous copper(II) complex (Saleh Salga *et al.*, 2010), the cadmium(II) ion is penta-coordinated by the *N,N',N"*-tridentate Schiff base ligand and two Cl atoms in a distorted square-pyramidal geometry, the τ value (Addison *et al.*, 1984) being 0.17. This arrangement is similar to what was observed in the structure of [CdCl_2_(C_10_H_15_N_3_)], the closest analogous cadmium complex (Bian *et al.*, 2003). In the crystal, pairs of the molecules, related by symmetry -*x*, -*y*, -*z* + 1, are bonded into centrosymmetric dimers *via* C7—H7B···Cl2 interaction.

### Experimental

A mixture of 2-acetylpyridine (0.61 g, 5 mmol) and *N*,*N*-dimethylethyldiamine (0.44 g, 5 mmol) in ethanol (50 ml) was refluxed for 2 hr followed by addition of a solution of cadmium(II) chloride (0.92 g, 5 mmol) in a minimum amount of water. The resulting solution was refluxed for 30 min, then set aside at room temperature. The colorless crystals of the title compound were obtained after a few days.

### Refinement

Hydrogen atoms were placed at calculated positions at distances C—H = 0.95, 0.98 and 0.99 ° for aryl, methyl and methylene type H-atoms, respectively, and were treated as riding on their parent atoms, with *U*_iso_(H) = 1.2–1.5 times *U*_eq_(C).

### Crystal data

**Table:**

[CdCl_2_(C_11_H_17_N_3_)]	*Z* = 2
*M**_r_* = 374.58	*F*(000) = 372
Triclinic, *P*1	*D*_x_ = 1.718 Mg m^−^^3^
Hall symbol: -P 1	Mo *K*α radiation, λ = 0.71073 Å
*a* = 8.0276 (2) Å	Cell parameters from 5105 reflections
*b* = 9.6048 (2) Å	θ = 2.2–29.7°
*c* = 10.0851 (2) Å	µ = 1.86 mm^−^^1^
α = 102.534 (1)°	*T* = 100 K
β = 103.365 (1)°	Plate, colorless
γ = 97.850 (1)°	0.23 × 0.11 × 0.04 mm
*V* = 724.09 (3) Å^3^	

### Data collection

**Table:**

Bruker APEXII CCD diffractometer	2652 independent reflections
Radiation source: fine-focus sealed tube	2547 reflections with *I* > 2σ(*I*)
graphite	*R*_int_ = 0.014
φ and ω scans	θ_max_ = 25.5°, θ_min_ = 2.2°
Absorption correction: multi-scan (*SADABS*; Sheldrick, 1996)	*h* = −9→9
*T*_min_ = 0.674, *T*_max_ = 0.929	*k* = −11→11
5023 measured reflections	*l* = −12→12

### Refinement

**Table:**

Refinement on *F*^2^	Primary atom site location: structure-invariant direct methods
Least-squares matrix: full	Secondary atom site location: difference Fourier map
*R*[*F*^2^ > 2σ(*F*^2^)] = 0.016	Hydrogen site location: inferred from neighbouring sites
*wR*(*F*^2^) = 0.038	H-atom parameters constrained
*S* = 1.08	*w* = 1/[σ^2^(*F*_o_^2^) + (0.0151*P*)^2^ + 0.4207*P*] where *P* = (*F*_o_^2^ + 2*F*_c_^2^)/3
2652 reflections	(Δ/σ)_max_ = 0.001
157 parameters	Δρ_max_ = 0.29 e Å^−^^3^
0 restraints	Δρ_min_ = −0.39 e Å^−^^3^

### Special details

**Table:**

Geometry. All e.s.d.'s (except the e.s.d. in the dihedral angle between two l.s. planes) are estimated using the full covariance matrix. The cell e.s.d.'s are taken into account individually in the estimation of e.s.d.'s in distances, angles and torsion angles; correlations between e.s.d.'s in cell parameters are only used when they are defined by crystal symmetry. An approximate (isotropic) treatment of cell e.s.d.'s is used for estimating e.s.d.'s involving l.s. planes.
Refinement. Refinement of *F*^2^ against ALL reflections. The weighted *R*-factor *wR* and goodness of fit *S* are based on *F*^2^, conventional *R*-factors *R* are based on *F*, with *F* set to zero for negative *F*^2^. The threshold expression of *F*^2^ > σ(*F*^2^) is used only for calculating *R*-factors(gt) *etc*. and is not relevant to the choice of reflections for refinement. *R*-factors based on *F*^2^ are statistically about twice as large as those based on *F*, and *R*- factors based on ALL data will be even larger.

### Fractional atomic coordinates and isotropic or equivalent isotropic displacement parameters (Å^2^)

**Table:**

	*x*	*y*	*z*	*U*_iso_*/*U*_eq_	
Cd1	−0.076971 (16)	0.190459 (13)	0.205336 (13)	0.01438 (5)	
Cl1	−0.19397 (6)	0.14396 (5)	−0.04976 (5)	0.02368 (11)	
Cl2	−0.21885 (7)	0.34705 (5)	0.35214 (5)	0.02337 (11)	
N1	−0.1880 (2)	−0.04027 (16)	0.23109 (16)	0.0155 (3)	
N2	0.1417 (2)	0.10346 (17)	0.33828 (16)	0.0171 (3)	
N3	0.1925 (2)	0.34724 (17)	0.22918 (16)	0.0187 (3)	
C1	−0.3543 (3)	−0.1088 (2)	0.1769 (2)	0.0193 (4)	
H1	−0.4351	−0.0611	0.1283	0.023*	
C2	−0.4143 (3)	−0.2472 (2)	0.1886 (2)	0.0214 (4)	
H2	−0.5330	−0.2941	0.1471	0.026*	
C3	−0.2973 (3)	−0.3147 (2)	0.2619 (2)	0.0233 (4)	
H3	−0.3346	−0.4091	0.2719	0.028*	
C4	−0.1247 (3)	−0.2432 (2)	0.3208 (2)	0.0202 (4)	
H4	−0.0429	−0.2874	0.3732	0.024*	
C5	−0.0729 (2)	−0.1063 (2)	0.30211 (19)	0.0166 (4)	
C6	0.1124 (2)	−0.0244 (2)	0.35613 (19)	0.0173 (4)	
C7	0.2517 (3)	−0.0968 (2)	0.4217 (2)	0.0244 (4)	
H7A	0.2968	−0.1510	0.3482	0.037*	
H7B	0.2025	−0.1639	0.4697	0.037*	
H7C	0.3470	−0.0229	0.4902	0.037*	
C8	0.3182 (2)	0.1896 (2)	0.3762 (2)	0.0212 (4)	
H8A	0.3840	0.1458	0.3117	0.025*	
H8B	0.3803	0.1910	0.4737	0.025*	
C9	0.3074 (3)	0.3439 (2)	0.3656 (2)	0.0211 (4)	
H9A	0.2626	0.3932	0.4431	0.025*	
H9B	0.4260	0.3985	0.3773	0.025*	
C10	0.2668 (3)	0.2957 (2)	0.1115 (2)	0.0246 (4)	
H10A	0.3822	0.3559	0.1282	0.037*	
H10B	0.1898	0.3027	0.0233	0.037*	
H10C	0.2779	0.1942	0.1045	0.037*	
C11	0.1656 (3)	0.4971 (2)	0.2359 (2)	0.0286 (5)	
H11A	0.2779	0.5613	0.2511	0.043*	
H11B	0.1150	0.5301	0.3140	0.043*	
H11C	0.0860	0.4995	0.1470	0.043*	

### Atomic displacement parameters (Å^2^)

**Table:**

	*U*^11^	*U*^22^	*U*^33^	*U*^12^	*U*^13^	*U*^23^
Cd1	0.01536 (8)	0.01317 (8)	0.01511 (8)	0.00330 (5)	0.00430 (5)	0.00417 (5)
Cl1	0.0233 (2)	0.0301 (3)	0.0159 (2)	0.0048 (2)	0.00258 (18)	0.00570 (19)
Cl2	0.0298 (3)	0.0205 (2)	0.0265 (2)	0.0112 (2)	0.0154 (2)	0.00792 (19)
N1	0.0182 (8)	0.0151 (7)	0.0148 (7)	0.0045 (6)	0.0055 (6)	0.0048 (6)
N2	0.0165 (8)	0.0180 (8)	0.0158 (8)	0.0040 (6)	0.0032 (6)	0.0031 (6)
N3	0.0202 (8)	0.0168 (8)	0.0194 (8)	0.0030 (6)	0.0076 (7)	0.0029 (6)
C1	0.0193 (10)	0.0203 (10)	0.0203 (9)	0.0059 (8)	0.0072 (8)	0.0063 (8)
C2	0.0200 (10)	0.0221 (10)	0.0230 (10)	0.0011 (8)	0.0091 (8)	0.0063 (8)
C3	0.0294 (11)	0.0165 (9)	0.0289 (11)	0.0041 (8)	0.0149 (9)	0.0084 (8)
C4	0.0264 (10)	0.0196 (10)	0.0211 (10)	0.0107 (8)	0.0114 (8)	0.0095 (8)
C5	0.0220 (10)	0.0162 (9)	0.0141 (9)	0.0075 (8)	0.0081 (7)	0.0033 (7)
C6	0.0205 (10)	0.0200 (9)	0.0129 (9)	0.0080 (8)	0.0056 (7)	0.0036 (7)
C7	0.0222 (10)	0.0253 (10)	0.0275 (11)	0.0083 (8)	0.0044 (9)	0.0107 (9)
C8	0.0169 (10)	0.0226 (10)	0.0218 (10)	0.0035 (8)	0.0028 (8)	0.0038 (8)
C9	0.0206 (10)	0.0194 (10)	0.0198 (10)	−0.0004 (8)	0.0057 (8)	−0.0001 (8)
C10	0.0239 (11)	0.0297 (11)	0.0202 (10)	0.0027 (9)	0.0100 (8)	0.0032 (8)
C11	0.0326 (12)	0.0171 (10)	0.0388 (12)	0.0030 (9)	0.0150 (10)	0.0083 (9)

### Geometric parameters (Å, °)

**Table:**

Cd1—N2	2.3188 (15)	C4—C5	1.390 (3)
Cd1—N1	2.3644 (15)	C4—H4	0.9500
Cd1—N3	2.3909 (16)	C5—C6	1.499 (3)
Cd1—Cl1	2.4434 (5)	C6—C7	1.497 (3)
Cd1—Cl2	2.4561 (5)	C7—H7A	0.9800
N1—C1	1.335 (2)	C7—H7B	0.9800
N1—C5	1.350 (2)	C7—H7C	0.9800
N2—C6	1.277 (2)	C8—C9	1.522 (3)
N2—C8	1.459 (2)	C8—H8A	0.9900
N3—C10	1.471 (2)	C8—H8B	0.9900
N3—C11	1.474 (2)	C9—H9A	0.9900
N3—C9	1.478 (2)	C9—H9B	0.9900
C1—C2	1.389 (3)	C10—H10A	0.9800
C1—H1	0.9500	C10—H10B	0.9800
C2—C3	1.379 (3)	C10—H10C	0.9800
C2—H2	0.9500	C11—H11A	0.9800
C3—C4	1.388 (3)	C11—H11B	0.9800
C3—H3	0.9500	C11—H11C	0.9800
			
N2—Cd1—N1	69.42 (5)	N1—C5—C6	115.96 (16)
N2—Cd1—N3	73.39 (5)	C4—C5—C6	122.77 (17)
N1—Cd1—N3	141.34 (5)	N2—C6—C7	123.96 (18)
N2—Cd1—Cl1	131.13 (4)	N2—C6—C5	116.71 (16)
N1—Cd1—Cl1	98.62 (4)	C7—C6—C5	119.29 (16)
N3—Cd1—Cl1	97.82 (4)	C6—C7—H7A	109.5
N2—Cd1—Cl2	112.49 (4)	C6—C7—H7B	109.5
N1—Cd1—Cl2	100.86 (4)	H7A—C7—H7B	109.5
N3—Cd1—Cl2	102.71 (4)	C6—C7—H7C	109.5
Cl1—Cd1—Cl2	116.315 (17)	H7A—C7—H7C	109.5
C1—N1—C5	119.09 (16)	H7B—C7—H7C	109.5
C1—N1—Cd1	124.01 (12)	N2—C8—C9	109.01 (16)
C5—N1—Cd1	116.87 (12)	N2—C8—H8A	109.9
C6—N2—C8	121.86 (16)	C9—C8—H8A	109.9
C6—N2—Cd1	120.70 (13)	N2—C8—H8B	109.9
C8—N2—Cd1	116.44 (11)	C9—C8—H8B	109.9
C10—N3—C11	110.06 (16)	H8A—C8—H8B	108.3
C10—N3—C9	111.56 (15)	N3—C9—C8	112.34 (15)
C11—N3—C9	108.99 (15)	N3—C9—H9A	109.1
C10—N3—Cd1	110.95 (11)	C8—C9—H9A	109.1
C11—N3—Cd1	110.21 (12)	N3—C9—H9B	109.1
C9—N3—Cd1	104.94 (11)	C8—C9—H9B	109.1
N1—C1—C2	122.72 (18)	H9A—C9—H9B	107.9
N1—C1—H1	118.6	N3—C10—H10A	109.5
C2—C1—H1	118.6	N3—C10—H10B	109.5
C3—C2—C1	118.40 (18)	H10A—C10—H10B	109.5
C3—C2—H2	120.8	N3—C10—H10C	109.5
C1—C2—H2	120.8	H10A—C10—H10C	109.5
C2—C3—C4	119.34 (18)	H10B—C10—H10C	109.5
C2—C3—H3	120.3	N3—C11—H11A	109.5
C4—C3—H3	120.3	N3—C11—H11B	109.5
C3—C4—C5	119.17 (18)	H11A—C11—H11B	109.5
C3—C4—H4	120.4	N3—C11—H11C	109.5
C5—C4—H4	120.4	H11A—C11—H11C	109.5
N1—C5—C4	121.26 (17)	H11B—C11—H11C	109.5

### Hydrogen-bond geometry (Å, °)

**Table:**

*D*—H···*A*	*D*—H	H···*A*	*D*···*A*	*D*—H···*A*
C7—H7B···Cl2^i^	0.98	2.77	3.679 (2)	155

Symmetry codes: (i) −*x*, −*y*, −*z*+1.
